# Early changes of ganglion cell-inner plexiform layer thickness and macular microvasculature in Posner-Schlossman syndrome: a binocular control study by OCTA

**DOI:** 10.3389/fmed.2023.1169504

**Published:** 2023-07-24

**Authors:** Zhiyi Hu, Liwei Zhu, Junli Xu, Jiamin Wei, Shuangqing Wu, Qi Dai, Qibin Xu

**Affiliations:** ^1^The Second Clinical Medical College, Zhejiang Chinese Medical University, Hangzhou, Zhejiang, China; ^2^Department of Ophthalmology, Zhejiang Medicine and Western Medicine Integrated Hospital, Hangzhou Red-Cross Hospital, Hangzhou, Zhejiang, China; ^3^National Clinical Research Center for Ocular Diseases, Eye Hospital, Wenzhou Medical University, Wenzhou, Zhejiang, China

**Keywords:** Posner-Schlossman syndrome (PSS), optical coherence tomography angiography (OCTA), macular ganglion cell-inner plexiform layer (mGCIPL), macular superficial microvasculature, vessel density (VD), perfusion density (PD)

## Abstract

To evaluate the early changes in ganglion cell-inner plexiform layer thickness and macular microvasculature in Posner-Schlossman syndrome (PSS) with a binocular control study involving optical coherence tomography angiography (OCTA). Twenty-six patients with unilateral PSS were included in this cross-sectional study. All subjects underwent a thorough ocular examination. Macular ganglion cell-inner plexiform layer (mGCIPL) and superficial macular microvasculature measurements, including vessel density (VD), perfusion density (PD) and the foveal avascular zone (FAZ), were recorded. In PSS-affected eyes, the mGCIPL thickness was significantly lower in all quadrants than in the contralateral eyes (all *p* < 0.05). Significant macular microvascular damage was found in the PSS-affected eyes, including whole-image VD (wiVD), wiPD, perifoveal VD (periVD) and periPD (all *p* < 0.05); but there was no obvious difference in parafoveal VD (paraVD), paraPD and FAZ parameters (all *p* > 0.05). In addition, a decreased wiVD and wiPD were significantly correlated with a smaller mGCIPL thickness and a decreased MD (all *p* < 0.05). These parameters may contribute to the early detection of glaucomatous damage and timely supervision of disease progression in PSS.

## 1. Introduction

Posner-Schlossman syndrome (PSS) was first proposed by Posner and Schlossman (1) as a special form of mild nongranulomatous anterior uveitis accompanied by elevated intraocular pressure that usually affects the unilateral eye. In the past, PSS was considered a self-limited, benign disease because the appearance of the optic disc was usually normal or reversible (2–4), and vision was rarely defective (1, 3, 4). However, an increasing number of studies have reported that in the long-term, recurrent episodes may ultimately lead to glaucomatous optic nerve damage and/or visual field impairment in PSS patients (5–7).

Glaucoma is an optic neuropathy characterized by the death of retinal ganglion cells (RGC) and their axons (8), more than 50% of which are in the macula (9). Recent studies have shown that macular parameters such as macular ganglion cell-inner plexiform layer (mGCIPL) thickness are better than or at least as effective as retinal nerve fiber layer (RNFL) parameters in the evaluation of RGC damage (10–12). Damage to macular RGC can be detected in the early stage of glaucoma, but there are few reports on the mGCIPL in PSS.

Optical coherence tomography angiography (OCTA) is a new technique for detecting changes in blood flow that can be used to quantitatively analyze retinal microvessels (13). Because RGCs and their axons lie in the inner retina, which is nourished by the superficial microcirculation (14, 15), superficial macular microvasculature parameters such as macular vessel density (VD), perfusion density (PD) and foveal avascular zone (FAZ) are considered more likely to reflect glaucomatous damage than deeper parameters (16–18).

To avoid underestimating the optic nerve damage caused by PSS, early detection and timely supervision are crucial for reducing disease progression. Based on the characteristics of the unilateral onset of PSS, the current study aimed to investigate the potential early changes in macular OCTA metrics by comparing mGCIPL, VD, PD and FAZ parameters between PSS-affected eyes and their fellow eyes.

## 2. Materials and methods

### 2.1. Participants

This is a cross-sectional, observational study. The research process is in line with the tenets of the Declaration of Helsinki, and this study was approved by the ethics committee of Zhejiang Integrated Traditional Chinese and Western Medicine Hospital. All patients were informed of the purpose and steps of the study and signed the written informed consent forms. A consecutive series of patients diagnosed with PSS and examined at our hospital were recruited for the study from November 2020 to December 2021.

The inclusion criteria were as follows: (1) unilateral onset PSS in the remission stage; (2) transient episodes of elevated intraocular pressure; (3) mild anterior chamber inflammation, open anterior chamber angle without iris synechiae, hoar and white suet-shaped keratic precipitates (KPs); (4) no posterior inflammation; and (5) visual field mean deviation (MD) ≥ −6 decibels (dB).

The exclusion criteria were as follows: (1) bilateral disease or bilateral alternate disease; (2) visual field MD < −6 dB; (3) uncontrolled intraocular pressure after drug treatment; (4) primary glaucoma or elevated intraocular pressure caused by other known factors; (5) previous history of ophthalmic surgery; (6) severe corneal opacity or cataract may affect the quality of fundus imaging; (7) history of retinal diseases; and (8) other systemic diseases and drug use, mental disorders or other reasons for not cooperating with the study.

### 2.2. Ocular examinations

All patients underwent complete ophthalmologic examinations, including best-corrected visual acuity (BCVA), refractive, intraocular pressure measurement with Goldmann applanation tonometry, slit-lamp biomicroscopy, indirect fundus ophthalmoscopy, visual acuity and OCTA examination.

The visual field test was performed by standard automated perimetry (SAP; Humphrey C24-2 SITA-Standard visual field; Zeiss Meditec, Dublin, United States). The reliability criteria of the visual field examination were fixation losses < 20%, and false-positive and false-negative errors < 15%; the perimeter software calculated the MD and pattern standard deviation (PSD). Early glaucoma was defined as a 24–2 visual field MD ≥ −6 dB.

All patients underwent macular angiography with a 6 × 6 mm scan using the Zeiss Cirrus 5,000 system (Carl Zeiss Meditec, Dublin, United States) to obtain microvasculature images of the macular areas. This instrument operates at a central wavelength of 840 nm and a speed of 68,000 A-scans per second and is equipped with a real-time eye-tracking system to reduce motion artifacts. The 6 × 6 scan pattern has 245 A-scans in each B-scan along both the horizontal and vertical dimensions. Vascular images of the superficial capillary plexus (from the internal limiting membrane to the inner plexiform layer) were displayed automatically.

**Figure 1:**
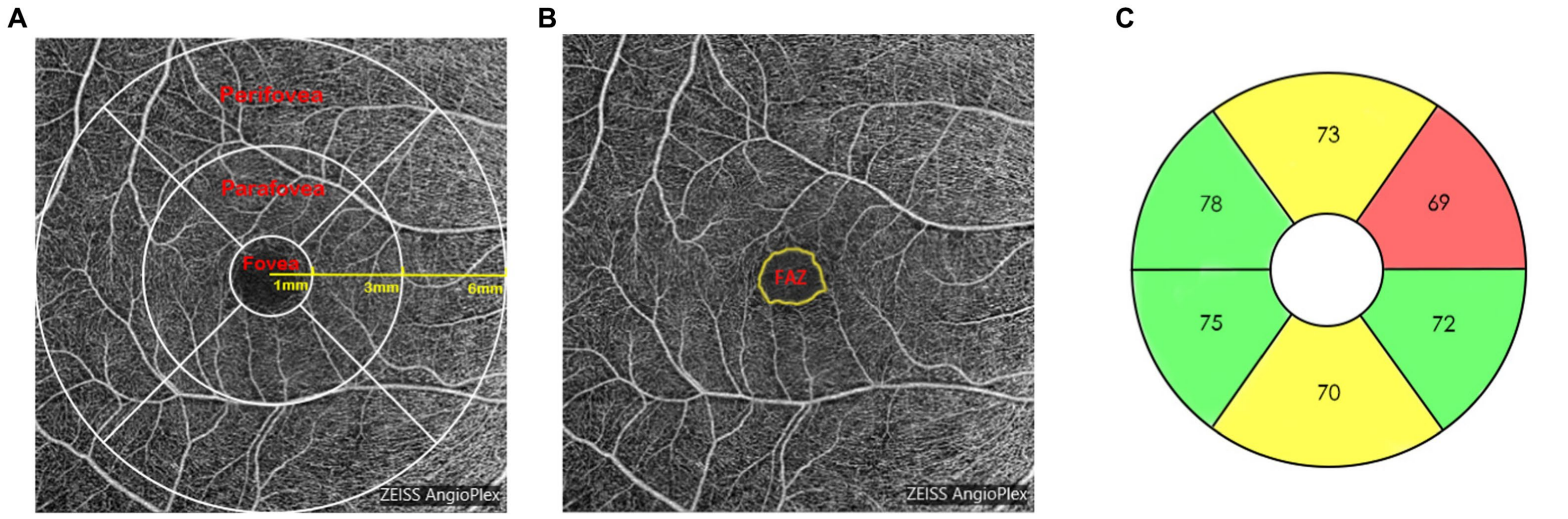
**(A)** Macular superficial vessel density and perfusion density measurement map with areas corresponding to foveal, parafoveal and perifoveal measurements; **(B)** automatically detected foveal avascular zone measurement map; **(C)** macular ganglion cell-inner plexiform layer measurement map with areas corresponding to the six sectors (superotemporal, superior, superonasal, inferonasal, inferior and inferotemporal).

All scans were analyzed using Cirrus OCTA software (AngioPlex, V.10.0). Vessel density (VD) was defined as the total length of perfused vasculature per unit area in a region of measurement, and perfusion density (PD) was defined as the total area of perfused vasculature per unit area in the region of measurement. According to the Early Treatment Diabetic Retinopathy Study, each 6 × 6 mm scan was automatically divided into three sectors to assess VD and PD parameters (Figure 1A): fovea (1 mm central circle), parafovea (3 mm inner circle), and perifovea (6 mm outer circle), as well as the whole scan area. The area, perimeter, and circularity (calculated as 4πA/P, where A is the area and P is the perimeter) of the foveal avascular zone (FAZ) were also measured (Figure 1B).

A macular cube with 512 × 128 scan protocols was used to measure the mGCIPL thickness. Within a 14.13 mm^2^ elliptical area centered on the fovea, the mGCIPL thickness was measured using a ganglion cell analysis algorithm that automatically divided the fovea into six sectors (superotemporal, superior, superonasal, inferonasal, inferior, and inferotemporal; Figure 1C), and the minimum and average mGCIPL thicknesses were analyzed.

All scans were performed by the same experienced operator (JMW) on the same instrument, and all scans were reviewed individually by one experienced examiner (JLX). Only images with signal strength (SS) ≥ 8 were included for analysis.

### 2.3. Statistical analyses

Statistical analyses were performed using SPSS software, version 26.0 (IBM Corp, Armonk, NY, United States). Data are presented as the means ± standard deviations. The Kolmogorov–Smirnov test was used to check the normality of the distribution of the data. Paired *t*-tests were used to compare the mGCIPL thickness and macular microvasculature parameters between PSS-affected eyes and their fellow eyes. Pearson’s correlation and univariate linear regression analysis were performed to analyze the correlation between macular microvasculature parameters, mGCIPL thickness, MD and other potential factors. *p* < 0.05 was considered to indicate statistical significance.

## 3. Results

### 3.1. Patient characteristics

Twenty-six patients fit the inclusion and exclusion criteria. Table 1 summarizes the demographic and clinical characteristics of all of the subjects. In our study, 5 patients had their first attack, and the others had a history of several PSS attacks. The average disease duration was 3.39 ± 6.56 years. The average peak IOP was 44.85 ± 10.72 mmHg. The mean deviation of the visual field of the PSS-affected eyes was −2.31 ± 2.03 dB.

**Table 1:** Demographic data, clinical findings and visual field properties (*n* = 26).

	Value
Age, years	43.64 ± 16.26 (17, 66)
Onset age, years	39.48 ± 17.02 (11, 64)
Sex (male/female)	16/10
Affect eye (OD/OS)	12/14
BCVA, decimal	0.98 ± 0.15 (0.8, 1.2)
Peak IOP, mmHg	44.85 ± 10.72 (29.30, 63.70)
Disease duration, years	3.39 ± 6.56 (1, 30)
Disease frequency, times per year	1.96 ± 1.12 (1, 4)
MD, dB	−2.31 ± 2.03 (−4.94, 0.93)
PSD, dB	1.35 ± 2.54 (0.44, 3.66)

BCVA, best-corrected visual acuity; IOP, intraocular pressure; MD, mean deviation; PSD, pattern standard deviation.

### 3.2. Comparison of macular GCIPL thickness

The mGCIPL thickness on average and in all quadrants was significantly lower in the PSS-affected eyes than in the fellow eyes (all *p* < 0.05; Table 2).

**Table 2:** Comparison of mGCIPL between PSS-affected eyes and their fellow eyes (*n* = 26).

	Affected Eye	Fellow Eye	Difference	*P*
**mGCIPL thickness (μm)**				
Average	72.31 ± 12.34 (60, 93)	80.96 ± 7.91 (68, 99)	8.65 (4.38, 12.93)	<0.001
Minimum	64.31 ± 16.30 (54, 90)	77.08 ± 7.28 (65, 91)	12.77 (5.43, 20.11)	0.001
Superior	72.38 ± 16.18 (56, 93)	81.69 ± 9.80 (68, 112)	9.31 (3.13, 15.48)	0.004
Superonasal	72.77 ± 16.33 (56, 92)	81.62 ± 9.12 (67, 102)	8.85 (2.55, 15.14)	0.009
Superotemporal	74.31 ± 12.18 (62, 97)	82.73 ± 8.56 (70, 102)	8.42 (3.82, 13.03)	0.001
Inferior	72.54 ± 14.01 (58, 89)	80.96 ± 7.83 (68, 92)	8.42 (3.50, 13.35)	0.002
Inferonasal	73.35 ± 14.29 (58, 92)	79.85 ± 8.03 (69, 95)	6.50 (0.97, 12.03)	0.025
Inferotemporal	72.42 ± 11.18 (57, 93)	81.77 ± 9.27 (67, 113)	9.35 (5.11, 13.58)	<0.001

PSS, Posner-Schlossman syndrome; mGCIPL, macular ganglion cell-inner plexiform layer. The paired t test was used to compare PSS-affected eyes and their fellow eyes. Bold values indicate statistical significance.

### 3.3. Comparison of macular microvasculature

As shown in Table 3, the whole-image of macular VD (wiVD) and the perifoveal VD (periVD) were significantly lower in PSS-affected eyes than in the fellow eyes (*p* = 0.030, *p* = 0.018, respectively). However, the foveal VD (fVD) and parafoveal VD (paraVD) showed no significant differences between PSS-affected eyes and fellow eyes (*p* > 0.05).

**Table 3:** Comparison of macular microvasculature between PSS-affected eyes and their fellow eyes (*n* = 26).

	Affected Eye	Fellow Eye	Difference	*P*
**Vessel density (mm** ^**−1**^ **)**				
wiVD	15.65 ± 3.21 (13.7, 19.1)	17.20 ± 1.96 (14.7, 19.1)	1.55 (0.16, 2.94)	0.030
fVD	7.67 ± 1.72 (5.1, 14.4)	8.47 ± 2.99 (6.0, 14.4)	0.79 (−0.42, 2.01)	0.193
paraVD	16.15 ± 3.27 (14.3, 19.7)	17.16 ± 2.59 (15.0, 19.2)	1.01 (−0.58, 2.59)	0.202
periVD	15.81 ± 3.38 (13.6, 19.2)	17.53 ± 1.97 (15.1, 19.4)	1.73 (0.32, 3.14)	0.018
**Perfusion density (%)**				
wiPD	38.03 ± 8.21 (33.6, 46.4)	41.57 ± 4.59 (37.9, 46.4)	3.97 (0.43, 7.51)	0.029
fPD	16.79 ± 4.27 (10.8, 24.5)	19.03 ± 7.28 (12.4, 28.2)	2.24 (−0.89, 5.37)	0.159
paraPD	38.16 ± 7.78 (33.9, 45.9)	40.44 ± 6.50 (34.4, 46.8)	2.28 (−1.68, 6.23)	0.247
periPD	37.60 ± 8.14 (30.6, 47.7)	41.58 ± 4.59 (36.3, 47.7)	4.00 (0.39, 7.61)	0.031
**Foveal avascular zone**				
Area (mm^2^)	0.239 ± 0.111 (0.11, 0.38)	0.254 ± 0.104 (0.11, 0.38)	0.015 (−0.024, 0.053)	0.440
Perimeter (mm)	2.012 ± 0.589 (1.55, 2.96)	2.062 ± 0.369 (1.55, 2.72)	0.049 (−0.164, 0.262)	0.638
Circularity	0.708 ± 0.105 (0.61, 0.80)	0.716 ± 0.095 (0.55, 0.82)	0.008 (−0.039, 0.055)	0.726

PSS, Posner-Schlossman syndrome; wiVD, whole-image vessel density; fVD, foveal vessel density; paraVD, parafoveal vessel density; periVD, perifoveal vessel density; wiPD, whole-image perfusion density; fPD, foveal perfusion density; paraPD, parafoveal perfusion density; periPD, perifoveal perfusion density. A paired *t*-test was used in PSS-affected eyes and PSS-fellow eyes. Bold values indicate statistical significance.

The macular PD followed the same trend as the VD. The foveal PD (fPD) and parafoveal PD (paraPD) were similar in PSS-affected eyes and their fellow eyes (*p* > 0.05). However, the whole-image PD (wiPD) and perifoveal PD (periPD) in PSS-affected eyes were lower than those in the fellow eyes (*p* = 0.029 in wiPD, and *p* = 0.031 in periPD).

For the FAZ parameters, there were no significant differences in the area, perimeter or circularity between PSS-affected eyes and their fellow eyes (all p > 0.05).

### 3.4. Correlation analysis of macular microvasculature with potential factors

**Figure 2:**
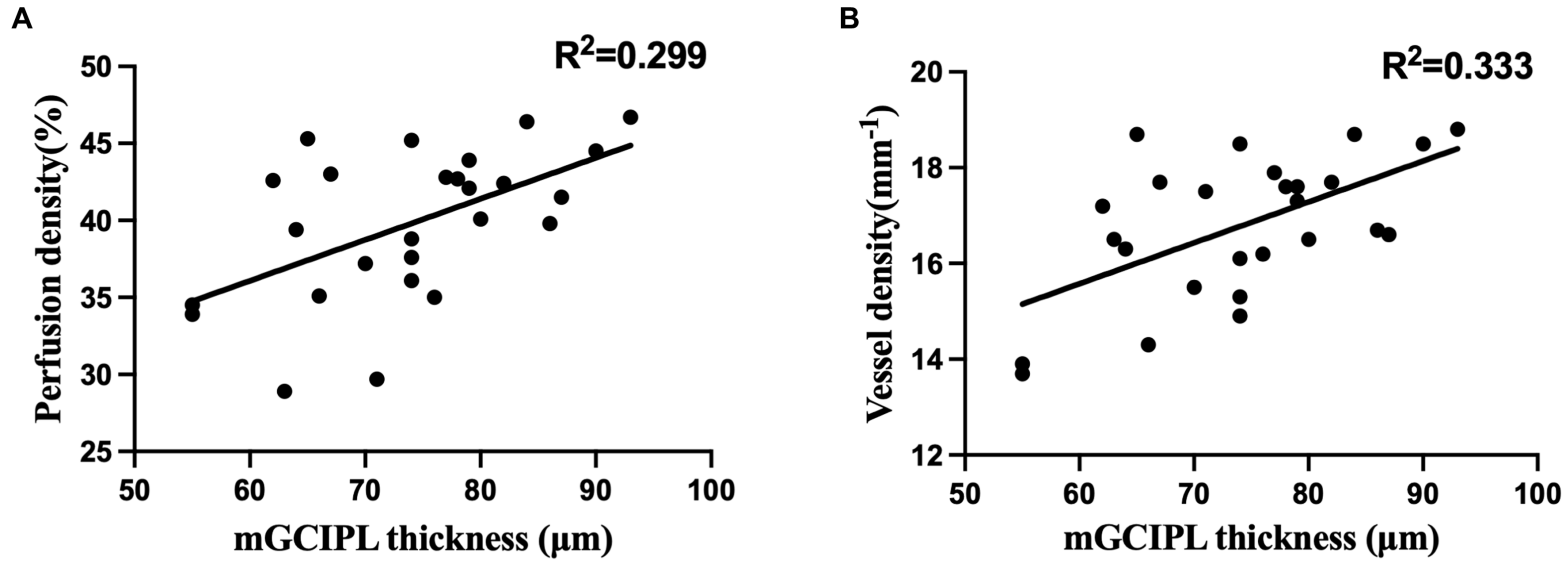
**(A)** Correlation between macular vessel density and mGCIPL thickness; **(B)** Correlation between macular perfusion density and mGCIPL thickness.

Table 4 shows the correlation of macular vascular parameters with mGCIPL thickness, MD and other potential factors in PSS-affected eyes. We found that both the wiVD (*p* = 0.049, r^2^ = 0.221) and wiPD (*p* = 0.050, r^2^ = 0.219) were positively correlated with the MD. Both the wiVD (*p* = 0.002, r^2^ = 0.333) and wiPD (*p* = 0.004, r^2^ = 0.299) showed a statistically significant correlation with the average mGCIPL thickness (Figure 2). However, neither the wiVD nor wiPD were associated with age, sex, onset age, disease duration, disease frequency, PD or peak IOP (all *p* > 0.05).

**Table 4:** Linear regression analysis for macular VD and PD in PSS-affected eyes.

Variables	Macular vessel density		Macular perfusion density	
	Coefficient (95%)	*P*	Coefficient (95%)	*P*
Age	−0.037 (−0.121, 0.046)	0.364	−0.090 (−0.302, 0.122)	0.390
Sex	−0.260 (−3.023, 2.503)	0.847	−0.343 (−7.360, 6.674)	0.920
Onset age	−0.909 (−3.287, 1.469)	0.436	−0.355 (−1.289, 0.579)	0.439
Disease duration	0.037 (−0.187, 0.261)	0.734	0.114 (−0.455, 0.682)	0.682
Disease frequency	−0.090 (−0.224, 0.045)	0.182	−0.037 (−0.089, 0.016)	0.164
MD	0.837 (0.004, 1.670)	**0.049**	2.122 (0.000, 4.244)	**0.050**
PSD	0.426 (−0.302, 1.155)	0.231	1.064 (−0.807, 2.934)	0.224
Peak IOP	−0.138 (−0.360, 0.083)	0.191	−0.341 (−0.909, 0.226)	0.207
mGCIPL thickness	3.896 (1.572, 6.220)	**0.002**	1.125 (0.400, 1.849)	**0.004**

PSS, Posner-Schlossman syndrome; IOP, intraocular pressure; mGCIPL, macular ganglion cell-inner plexiform layer; MD, mean deviation; PSD, pattern standard deviation. Bold values indicate statistical significance.

## 4. Discussion

PSS is no longer a self-limiting benign disease (5–7). Due to the mild nature of the symptoms, patients might suffer multiple episodes of ocular hypertension and therefore develop optic nerve damage before presenting to a specialist. In recent years, technology has developed that makes high-resolution retinal imaging possible, and several papers have suggested the importance of evaluating the retinal layer thickness to better understand the physiopathology of ocular, neurological or systemic diseases (19, 20). In an effort to provide a more sensible tool for detecting early glaucomatous change, we used the mGCIPL thickness and found that the PSS-affected eyes exhibited a significant decrease on average and in all quadrants compared with the PSS-fellow eyes, indicating the death of a great number of RGC and loss of their axons. This pattern is in accordance with other types of early glaucoma (10, 11), suggesting that PSS may be driven by a pathogenesis similar to that of other types of glaucoma.

Decreased macular superficial VD and PD were found in PSS-affected eyes relative to their fellow eyes (21). However, the early changes in the macular microvasculature in PSS has not been clearly elucidated. In the current study, we found that the wiVD, wiPD, periVD, and periPD were significantly decreased in PSS-affected eyes compared with PSS-fellow eyes, while no significant difference was found in the fVD, fPD, paraVD, and paraPD. This result suggests that the perifoveal microcirculation is more vulnerable to glaucomatous damage in the early stage of PSS. The same result was also found in both pre-perimetric glaucoma (22) and mild primary open-angle glaucoma (23), in which the periVD was more obviously damaged. In addition, we demonstrated that there were no significant differences in the FAZ area, perimeter, and circularity between PSS-affected eyes and their fellow eyes. There are a few possible hypotheses for these results. First, the FAZ is a small region in the fovea, which lacks retinal vessels, so slight changes in the microvasculature may be imperceptible (21). Second, the fovea contains only photoreceptor cells, which are usually not directly affected by glaucoma (24). Third, Van Melkebeke et al. (25) believed that the macular region most prone to glaucoma was primarily located outside the central 3 × 3 mm region but within the 6 × 6 mm region. Taken together, the results of our study correspond to previous studies suggesting that glaucomatous vascular injury might begin in the perifoveal region, progress to the parafoveal region and finally affect the FAZ parameters (21, 22, 26, 27).

We found that the average mGCIPL thickness in the PSS-affected eyes was correlated well with the wiVD and wiPD. Similar results can be found in the early stage of other types of glaucoma (17, 28). Kim et al. (17) maintained that because RGC were less affected, the relationship between mGCIPL thickness and the VD was more linear than that in advanced-stage glaucoma. These findings indicate a strong association between decreased microvasculature and RGC loss.

It has long been debated whether microvascular damage or RGC loss occurs first in glaucoma patients. Recent studies have shown that microvascular changes occur after RGC damage in glaucoma (22, 29–32). They therefore consider the decrease in microvasculature to be the result of capillary closure following RGC death. Moreover, Lu et al. (22) reported a topographic relationship between macular microvasculature damage and macular RGC loss in early primary open-angle glaucoma. In the current study, mGCIPL thickness was significantly lower in all quadrants, while obvious vascular damage occurred only in the perifoveal area in the early stage of PSS. In this sense, macular microvasculature changes may occur after mGCIPL damage in the pathogenesis of PSS. Further studies are necessary to better verify this finding.

Additionally, although the patients in our study did not have a severely impaired visual field (MD ≥ −6), we still found that both the wiVD and wiPD were correlated with MD, suggesting a correlation between macular vascular parameters and visual functional parameters. Several studies have proven that the reduction in the VD corresponds with the severity of visual field damage and with the severity of glaucoma (33–35). Thus, the evaluation of VD and PD loss could potentially be used to estimate visual function in the early stage of PSS.

Jap et al. (5) held that disease duration was the only factor leading to glaucomatous optic nerve injury in PSS. Gao et al. (36) showed that both the duration of the disease and the frequency of attacks affected the progression of PSS. However, we could not find the correlation between disease duration or disease frequency and the wiVD or wiPD in our study. The most important reason may be due to the different inclusion criteria in our research. We only selected patients with early visual field defects, excluding patients with middle and late visual field defects, or patients underwent glaucoma filtering surgery, which is different from the sample selection in the above paper (5, 36). Accordingly, the average course of disease in our patients was 3.39 ± 6.56 years, while was significantly shorter than 9.5 ± 10.4 years in Gao’s study (36). The selection bias and a relatively small sample size may be responsible for the different results in our study from previous studies (5, 36). A longitudinal study with larger sample size is needed to further investigate the influencing factors on the progression of PSS.

There are several other limitations to our study. First, the relatively small sample size limited the power of our findings; in addition, this study lacked some factors that may affect macular microvasculature including systemic vascular diseases such as hypertension. Finally, further longitudinal studies will better elucidate the correlation between macular microvasculature and visual function or RGC damage over time in the course of PSS progression.

## 5. Conclusion

In conclusion, the macular structural and vascular parameters were significantly lower in eyes with early-stage PSS than in the fellow eyes, and the vascular damage was more prominent in the perifoveal area. The reduced macular microvasculature parameters were correlated with a smaller mGCIPL thickness and decreased MD in the PSS-affected eyes. Binocular comparisons of the mGCIPL and macular microvasculature with OCTA may contribute to the early detection of glaucomatous damage and timely monitoring of disease progression in patients with PSS.

## Data availability statement

The datasets presented in this study can be found in online repositories. The names of the repository/repositories and accession number(s) can be found in the article/supplementary material.

## Ethics statement

The research process is in line with the tents of the Declaration of Helsinki, and this study has been approved by the Ethics Committee of Zhejiang Integrated Traditional Chinese and Western Medicine Hospital. All patients were informed of the purpose and steps of the study and signed the written informed consents.

## Author contributions

ZH, LZ, and QX analyzed and interpreted the data and wrote the manuscript. SW and QD contributed to statistical guidance and reviewed and approved the manuscript. SW, QD, and QX reviewed and approved the manuscript and designed the study. QX was the guarantor of the work, and had full access to all the data in the study, and takes responsibility for the integrity of the data and the accuracy of the data analysis. All authors contributed to the article and approved the submitted version.

## Conflict of interest

The authors declare that the research was conducted in the absence of any commercial or financial relationships that could be construed as a potential conflict of interest.

## Publisher’s note

All claims expressed in this article are solely those of the authors and do not necessarily represent those of their affiliated organizations, or those of the publisher, the editors and the reviewers. Any product that may be evaluated in this article, or claim that may be made by its manufacturer, is not guaranteed or endorsed by the publisher.
